# Comparative Metabolite Profiling of Two Rice Genotypes with Contrasting Salt Stress Tolerance at the Seedling Stage

**DOI:** 10.1371/journal.pone.0108020

**Published:** 2014-09-29

**Authors:** Xiuqin Zhao, Wensheng Wang, Fan Zhang, Jianli Deng, Zhikang Li, Binying Fu

**Affiliations:** Institute of Crop Sciences, National Key Facility for Crop Gene Resources and Genetic Improvement, Chinese Academy of Agricultural Sciences, Beijing, China; International Rice Research Institute, Philippines

## Abstract

**Background:**

Rice is sensitive to salt stress, especially at the seedling stage, with rice varieties differing remarkably in salt tolerance (ST). To understand the physiological mechanisms of ST, we investigated salt stress responses at the metabolite level.

**Methods:**

Gas chromatography-mass spectrometry was used to profile metabolite changes in the salt-tolerant line FL478 and the sensitive variety IR64 under a salt-stress time series. Additionally, several physiological traits related to ST were investigated.

**Results:**

We characterized 92 primary metabolites in the leaves and roots of the two genotypes under stress and control conditions. The metabolites were temporally, tissue-specifically and genotype-dependently regulated under salt stress. Sugars and amino acids (AAs) increased significantly in the leaves and roots of both genotypes, while organic acids (OAs) increased in roots and decreased in leaves. Compared with IR64, FL478 experienced greater increases in sugars and AAs and more pronounced decreases in OAs in both tissues; additionally, the maximum change in sugars and AAs occurred later, while OAs changed earlier. Moreover, less Na^+^ and higher relative water content were observed in FL478. Eleven metabolites, including AAs and sugars, were specifically increased in FL478 over the course of the treatment.

**Conclusions:**

Metabolic responses of rice to salt stress are dynamic and involve many metabolites. The greater ST of FL478 is due to different adaptive reactions at different stress times. At early salt-stress stages, FL478 adapts to stress by decreasing OA levels or by quickly depressing growth; during later stages, more metabolites are accumulated, thereby serving as compatible solutes against osmotic challenge induced by salt stress.

## Introduction

Rice (*Oryza sativa* L.) is the staple food for nearly half of the world's population. However, its growth and yield are greatly influenced by soil salinity [1]–[2], especially at the seedling stage [3]–[4]. An understanding of the physio-biochemical attributes that enable plants to survive under saline conditions is beneficial for improving rice production.

A high cellular salt concentration leads to ionic and osmotic imbalance that disrupts plant ion homeostasis and water potential, resulting in metabolic damage, growth arrest and even death [5]. Significant differences in salt tolerance (ST) between varieties are mainly related to the ability of plants to regulate the amount of sodium (Na^+^) and chloride reaching leaves or to metabolic regulation when roots are presented with saline conditions [6]–[8]. Higher tolerance to elevated Na^+^ is also sometimes associated with these differences [9]. Compared with the effects of ion toxicity, rice is reported to suffer greater osmotic stress under saline conditions. The contents of several metabolites, including amino acids (AAs), sugars and polyols, are increased under saline conditions, thereby serving as a defense against osmotic challenge by acting as compatible solutes [10]–[15].

Metabolites are the end products of cellular regulatory processes. Their levels can be regarded as the ultimate response of biological systems to genetic or environmental changes and are closely associated with phenotype [16]. In rice, metabolic analysis of ST has been very limited relative to substantive transcriptomics and/or proteomics research [17]–[21]. To date, no reports have appeared on metabolic response to salt stress over time in varieties with different ST, even though the systematic analysis of metabolic snapshots is considered a valid approach for quantitative description of cellular regulation and control [16], [22]. Furthermore, contradictory views exist regarding the functions of some metabolites during stress response. For example, proline appears to be a preferred organic osmoticum in many plants [10], [12], [23]–[26], whereas proline accumulation in rice is considered to be a symptom of injury rather than an indicator of ST [27]–[29].

In the current study, we used GC-MS to conduct metabolic profiling analysis on leaves and roots of two rice varieties, FL478 and IR64, under control and saline conditions. We also simultaneously investigated morphological and physiological traits. The two varieties differ greatly in ST and in their metabolic responses to the imposed salt stress. Our results provide insights into the specific adaptive response of rice to salt stress.

## Material and Methods

Two contrasting rice genotypes, FL478 and IR64, were used as research materials. FL478 is a salt-tolerant recombinant inbred line developed at the International Rice Research Institute using Pokkali (a salt-tolerant donor) and IR29. Salt-sensitive IR64 is a commercial variety from Asia.

### Plant growth conditions

Pot experiments, designed to investigate the different metabolic, morphological and physiological responses to salt stress between FL478 and IR64, were conducted in a greenhouse at the Chinese Academy of Agricultural Science, Beijing, China, in May 2012. After establishing water screens, the average daytime temperature in the greenhouse was about 25–29°C, which was appropriate for rice growth. The rice plants were cultivated in nutrient solution [30]. Salt treatments were conducted as reported previously [31]. Briefly, rice plants were cultivated to the four-leaf stage, and stress was then applied by adding NaCl to a final concentration of 100 mM and an electrical conductivity (EC) of 12 dS m^−1^. The EC of the control tanks was around 1.0 dS m^−1^. Three replicates were performed per treatment.

### Physiological trait analysis

#### Salt score (SS)

Salt scoring was conducted 7 d after the stress treatment. The salt stress symptoms of 10 plants per line were scored according to a modified standard evaluation system [32] for ST at the seedling stage, which ranged from 1 (normal growth) to 9 (almost all plants dead or dying).

#### Relative water content (RWC)

The youngest fully expanded leaves were harvested at 7 d for RWC analysis. RWC (%) was calculated as 100× (FLW − DLW)/(TLW − DLW), where FLW, TLW and DLW are fresh leaf weight, turgid leaf weight after saturation and dry leaf weight, respectively.

#### Na^+^ and K^+^ in shoots and roots

Na^+^ and K^+^ contents in both shoots and roots under control and saline conditions at 7 d were estimated using a flame photometer (S2; Thermo Finnigan, Waltham, MA, USA). After washing with distilled water to remove surface Na^+^ contamination, shoot and root samples were left to dry at 50°C for 4 d. The dried tissue was ground into a powder in liquid nitrogen. The resulting powder was acid digested by suspending in 5 ml of concentrated nitric acid overnight. Five milliliters of a 10∶4 diacid mixture of nitrate and perchlorate was added to the partially digested tissue powder, which was then incubated for 2 h on a sand bath to allow complete digestion. The digested solution was diluted to 25 ml with double-distilled water. Na^+^ and K^+^ levels in the acid-digested samples, representing total Na^+^ and K^+^ in the tissue samples, were estimated using the flame photometer.

#### Length and dry weight of shoots and roots

Plants grown under stress and control conditions were harvested at 7 d. Plant height, root length, and dry weight of the aboveground biomass and roots were measured.

### Metabolite extraction and identification

The topmost leaves and roots of plants cultivated under both control and stress conditions were collected simultaneously at 1, 3 and 7 d after salt treatment. The samples were flash-frozen in liquid nitrogen and stored at −70°C until metabolite extraction.

The samples were ground in liquid nitrogen with a pestle and mortar. Separate aliquots of the frozen, ground sample were used for metabolite extraction as described previously [33]–[34]. The extracted samples were then derivatized and analyzed by gas chromatography-mass spectrometry (GC-MS). The GC-MS system comprised an AOC 5000 auto-sampler, GC 2010 gas chromatograph and Voyager quadrupole mass spectrometer (Shimadzu, Kyoto, Japan). The mass spectrometer was tuned using tris-(perfluorobutyl)-amine. GC was performed on a 30-m Rtx-5 MS column with a 0.25-µm film thickness. A mixture of leaves and roots of both genotypes under stress and control conditions at 1, 3 and 7 d was extracted in bulk and used as a reference. Reference samples were run once every 10 samples. *N*-methyl-*N*-[trimethylsilyl] trifluoroacetamide (MSTFA) was run once every five samples to clean the injection surface of potential pollution.

Chromatograms and mass spectra were processed using the find algorithm implemented in GC-MS Postrun Analysis software (Shimadzu). Specific mass spectral fragments were detected in defined retention time windows using the mass spectral library NIST (http://www.nist.gov/mml/chemical_properties/data/) and the public domain mass spectral library of the Max Planck Institute for Plant Physiology, Golm, Germany (http://csbdb.mpimp-golm.mpg.de/). Most AAs, organic acids (OAs) and sugars were verified by performing standard addition experiments using pure authenticated compounds.

### Data analysis

The denominator of the quotient was the average response of the reference sample. The sample responses were volume-corrected with ribitol to compensate for errors during sample preparation or GC injection and normalized using sample fresh weight.

Analysis of variance (ANOVA) was performed in SAS v6.12 (SAS Institute Inc., Cary, NC, USA, 1996) was performed to determine the significance of trait differences between genotypes (*G*), tissues (*T*), sampling time points (*t*) and treatments (*S*). Specifically, metabolite differences associated with *G* and *T* were analyzed using data obtained at 1 d under control conditions, and metabolite differences related to *t* were analyzed with data from each genotype at three sampling time points under control conditions. Finally, metabolite differences due to *S* were analyzed using data from both control and stress treatments of each genotype at each time point. Differentially changed metabolites were defined as those showing significant concentration increases or decreases relative to their respective controls at *P*≤0.05 in ANOVA. Hierarchical cluster analysis of metabolites in roots and leaves was carried out using R software (http://www.r-project.org/).

## Results

### Salt stress effects on morphological and physiological traits of FL478 and IR64

**Figure 1:**
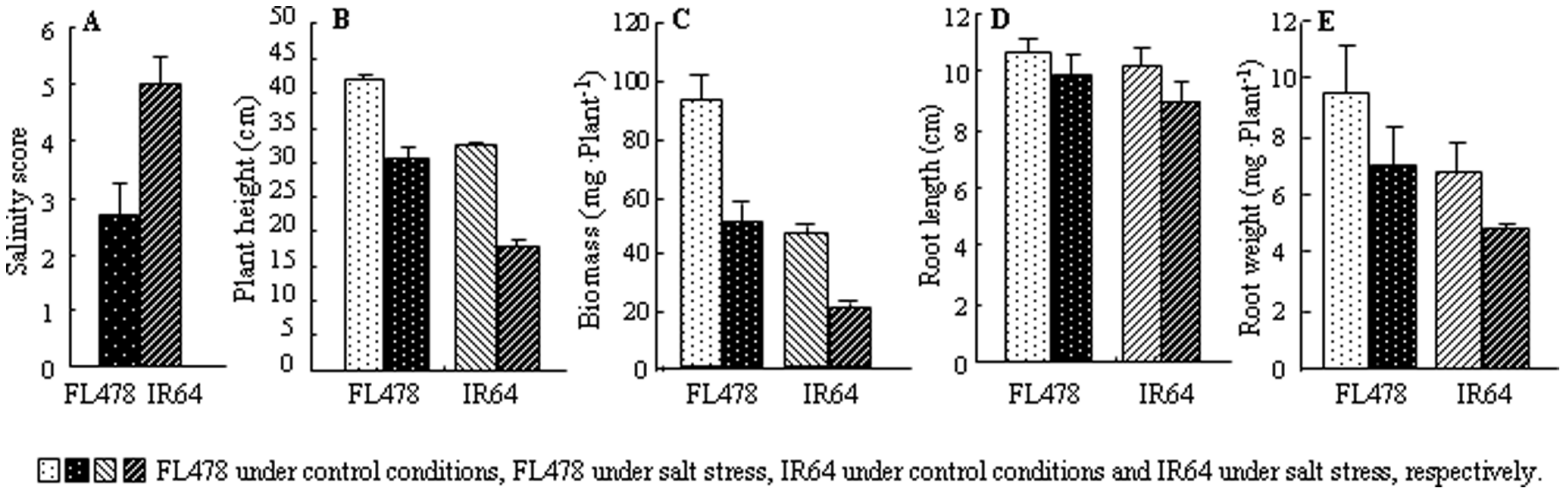
Morphological traits measured in both FL478 and IR64 under control and/or salt stress conditions. A: salinity score under salt conditions. B: plant height. C: plant biomass. D: root length. E: root dry weight.

The two genotypes, FL478 and IR64, were evaluated for growth parameters after the time-series salt treatment. As shown in Figure 1, the salinity score (SS) values of both genotypes altered under salt stress. FL478 had much better ST than IR64, as confirmed by a lower SS (SS_FL478_ = 2.67; SS_IR64_ = 5) (Figure 1A) and reduced repression of growth. For example, plant height and biomass respectively decreased by 27.6% and 46.7% in FL478 and 45.1% and 56.8% in IR64 (Figure 1B–C). Although salt stress had relatively less effect on root length and root dry weight (Figure 1D–E).

**Figure 2:**
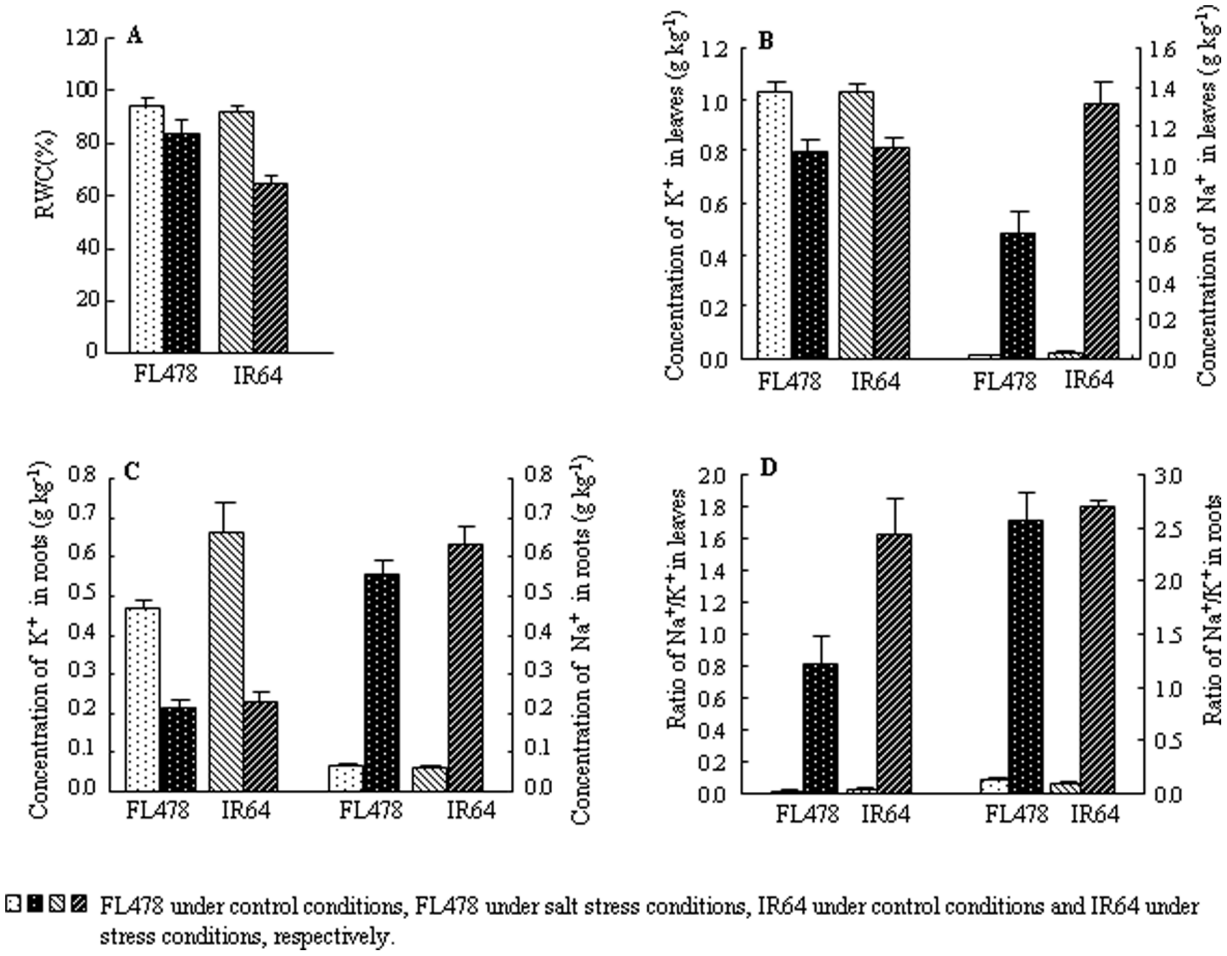
Physiological traits measured in both FL478 and IR64 under salt stress and control conditions. A: relative water content (RWC). B: K^+^ and Na^+^ concentration in leaves. C: K^+^ and Na^+^ concentration in roots. D: ratio of Na^+^/K^+^ in leaves and roots.

Physiological analysis revealed that RWC decreased by 29.9% in IR64 but only 11.7% in FL478 under salt stress (Figure 2A). Salt treatment drastically decreased the concentration of K^+^ and increased that of Na^+^ in both leaves and roots. Na^+^ concentration increased by 34.8- and 46.2-fold in leaves of FL478 and IR64, respectively (Figure 2B), and 7.7- and 9.1-fold in roots of FL478 and IR64, respectively (Figure 2C). The most significant Na^+^ increase was observed in leaves of IR64, with the Na^+^ concentration in leaves of IR64 about twice that of FL478 under saline conditions. Additionally, under salt stress, leaves of IR64 displayed higher Na^+^/K^+^ ratio levels than those of FL478; in roots, similar ratios were observed between the two genotypes (Figure 2D).

### Metabolite profiles of FL478 and IR64 under control and salt stress conditions

Using GC-MS, 89 and 86 metabolites were identified in leaves and roots, respectively, of both FL478 and IR64 (Table S1) under control and salt stress conditions.

Differences between genotypes (*G*), tissues (*T*) and sampling time points (*t*) were examined with ANOVA (Table S2). Out of all metabolites identified from both tissues, 30–32 metabolites (35–40%) showed significant *G* differences in different tissues at 1 d under control conditions; these differences, on average, explained 85.1–87.8% of the total phenotypic variation in these metabolites (Table S2.1). Additionally, 68–70 metabolites (74–76%) showed significant *T* differences in FL478 and IR64, respectively, which, on average, explained 92–95% of the total phenotypic variation (Table S2.2). Finally, 36–44 (44–49%) metabolites in leaves and 54–56 (63–65%) metabolites in roots were greatly influenced by sampling time, which, on average, explained 82.3–88.0% of the total phenotypic variation of the measured metabolites (Table S2.3). Because of the greater sensitivity of the metabolites to *t* and *T*, we focused our comparative study on individual metabolites showing significant differences under stress treatment in roots and leaves of both genotypes.

### An overview of metabolite changes in roots and leaves

**Figure 3:**
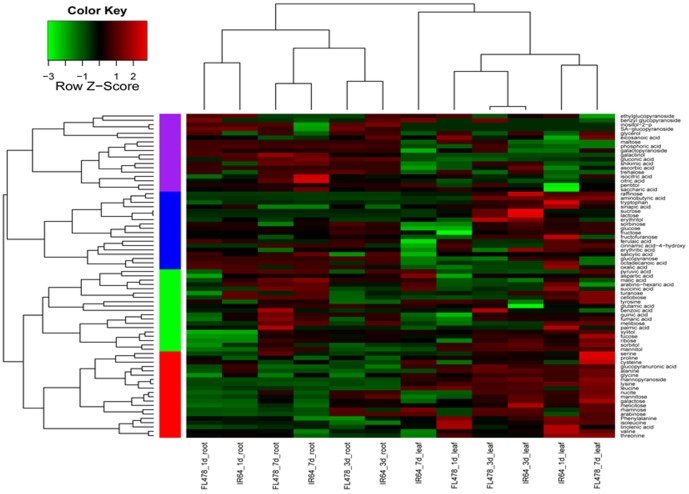
Results of hierarchical cluster analysis of changed metabolite pools. Hierarchical trees were drawn based on detected changed metabolites in leaves and roots of FL478 and IR64 under 1-, 3- and 7-d salt stress treatments. Columns correspond to genotypes at different time points, while rows represent different metabolites. Red and green colors indicate increased and decreased metabolite concentrations, respectively.

All metabolite change data from roots and leaves of both cultivars were analyzed by hierarchical clustering to provide a global view of metabolite changes in response to salt stress (Figure 3). Leaf and root samples were clustered into distinct groups. Clear separations were observed between root samples collected at different time points. In contrast, the clustering of leaf samples was more complicated: IR64 at 1 d and FL478 at 7 d clustered into one group, and IR64 at 3 d and FL478 at 3 d clustered into another, with IR64 at 7 d separated significantly from the other samples. These results indicate that differences in the metabolic phenotypes of sensitive and tolerant rice cultivars were more pronounced in leaves than in roots.

### Differentially changed metabolites in leaves of IR64 vs. FL478

To determine the responses of each genotype to salt stress, we compared levels of each metabolite in the stressed plants to levels in the control plants at similar time points (Table S3).

#### AA regulation under salt stress

Salt stress generally increased the levels of all 15 AAs in FL478 and IR64 (Table S3). Of the total 90 stress vs. control comparisons, 55 (61.1%) cases showed increases, whereas two (2.2%) decreased significantly. Compared with IR64, FL478 had more AAs showing increased levels. Moreover, the maximum number of measured AAs showing significantly increased levels occurred from 3–7 d in FL478, but occurred only at 3 d in IR64. The highest change levels occurred at 1 d in both genotypes. Only phenylalanine consistently increased in both genotypes over the course of the treatment. Five AAs (lysine, valine, proline, isoleucine and threonine) consistently increased in FL478 during salt treatment, with significant 3.25–6.91-fold increases observed; these same AAs were decreased or unchanged in IR64 at 7 d of salt treatment.

#### Sugar regulation under salt stress

Concentrations of most sugars were increased by salt treatment, indicating that increased sugar is beneficial to ST (Table S3). Out of 186 stress vs. control comparisons, 67 (36%) and 11 (5.9%) involved significant increases and decreases, respectively. Compared with IR64, FL478 had significantly more sugars showing increased levels. Additionally, the maximum number of sugars showing significantly increased levels occurred at 3–7 d in FL478 and 1–3 d in IR64. The highest change levels occurred at 7 d in FL478 and 3 d in IR64. Only melicitose and raffinose showed consistently increased levels across all time points of the stress treatment in both genotypes. Four sugars (sucrose, lactose, sorbitol and mannitol) were consistently increased over the course of the stress treatment only in FL478.

#### OA regulation under salt stress

Interestingly, in contrast to the change patterns observed for AAs and measured sugars, salt stress caused a decrease in most OAs in both IR64 and FL478 (Table S3). Of 156 stress vs. control comparisons, 21 (13.5%) involved increases while 31 (19.91%) exhibited significant decreases. Compared with IR64, FL478 had more OAs showing significantly decreased levels, especially at 1–3 d. OAs fumaric acid, succinic acid, malic acid and oxalic acid, involved in the tricarboxylic acid (TCA) cycle, decreased significantly in FL478, but increased or showed no change in IR64 at 1 d. Isocitric acid was specifically increased in FL478 over the course of the treatment.

### Differentially changed metabolites in roots of FL478 vs. IR64

We identified 86 compounds in roots. Table S4 shows the range of metabolites detected and how their levels changed after different stress treatment durations. Eighty of the 86 metabolites identified in roots were differentially regulated in both genotypes at least one salt stress time point. Similar to the change pattern of metabolites in leaves, most of the significantly changed metabolites were induced in roots. This result confirms that the metabolite increases were favorable for plant growth under saline conditions.

#### AA regulation in roots under salt stress

A number of AAs increased under salt stress. Out of 90 stress vs. control comparisons, 28 (31.1%) and 12 (13.3%) cases increased and decreased significantly, respectively (Table S4). Threonine showed consistently increased levels across all stress time points in both genotypes, while five AAs (phenylalanine, tryptophan, glutamic acid, aspartic acid and proline) showed increased levels at two time points in one or both genotypes. Compared with IR64, FL478 had a slightly larger number of increased AAs and a higher average change level, especially at 7 d. Proline levels increased only during stress treatment in FL478.

#### Sugar regulation in roots under salt stress

More sugars were observed to increase than to decrease in roots of both genotypes under saline conditions (Table S4). Out of 180 stress vs. control comparisons, 67 (37.2%) and 28 (15.6%) cases were associated with significant increases and decreases, respectively. Overall, similar change patterns were uncovered between the roots of the two genotypes, including similar change numbers, change levels and times associated with the maximum number of sugars showing significant variation (3 d in both rice genotypes). Nevertheless, change levels of sugars were generally lower in roots than in leaves over the course of the stress treatment. Only galactinol was commonly increased in both genotypes. Galactopyranoside was increased specifically in FL478 (Table S4).

#### OA regulation in roots under salt stress

Most OAs were differentially regulated in roots at at least one salt stress sampling point (Table S4). Interestingly, unlike the change pattern in leaves in which more OAs were decreased, a larger number of OAs increased in roots under salt stress. Out of 150 stress vs. control comparisons, 53 (35.3%) and 23 (15.3%) cases involved significant increases and decreases in roots, respectively. The maximum number of OAs showing significantly increased levels occurred at 7 d in both FL478 and IR64. Compared with IR64, FL478 had a greater number of decreased OAs across the three stress time points. These decreased OAs included two participants in the TCA cycle: fumaric and succinic acid. Only citric acid was commonly increased in both genotypes across the three sampling time points; however, FL478 had a much lower change level than IR64, with citric acid levels increasing 1.5–6.8-fold in FL478 and 2.0–12.3-fold in IR64.

## Discussion

Rice genotypes vary considerably in salt stress tolerance. We analyzed two rice genotypes, FL478 and IR64, with contrasting ST. Under high salt conditions, these two genotypes performed differently over the 7-d treatment. Compared with IR64, FL478 was less affected, as evidenced by a smaller reduction in biomass, plant height and root length, and a lower SS value. Shoot growth was more extensively affected than root growth in both genotypes, which is consistent with a previous report that leaf growth was more seriously affected by salt stress than root growth [35]. Moreover, a higher RWC was observed in FL478 under salt stress, a condition that may help rice plants maintain cell turgidity and normal metabolism under stress conditions.

Although salt stress caused remarkably decreased potassium and increased sodium levels in both genotypes, the extent of change was much lower in FL478. Under saline conditions, FL478 had a much lower Na^+^ concentration in leaves, i.e., nearly half of that found in IR64. At the same time, the Na^+^/K^+^ ratio was significantly lower in leaves of FL478 than in IR64. Similar Na^+^/K^+^ ratios were found between roots of the two genotypes (Figure 2D), demonstrating that less sodium was translocated from roots into FL478 leaves. All these results imply that the ST of FL478 is due to the capacity of this genotype to maintain a lower Na^+^/K^+^ ratio in leaves. This conclusion is in accord with previous studies [6]–[9].

GC-MS analysis revealed that most AAs and sugars increased significantly in leaves and roots of both genotypes under salt stress. Compared with IR64, the salt-tolerant variety FL478 had a later and more prolonged increase in AAs and sugars (Tables S3 and S4). This result is consistent with a previous study of barley under salt stress, in which an increase in AA levels occurred later in a salt-tolerant variety than in a sensitive variety [9]. AAs have been observed to generally increase in many plant species, where they act as osmolytes in response to abiotic stress [36] or are considered to be an indicator of general stress [9]. These increased AAs may be the result of *de novo* synthesis, reduced protein synthesis or general protein breakdown during stress [9], [36]–[39]. Nevertheless, the manner in which increased AA levels contribute directly or indirectly to salt stress tolerance is still unclear. Soluble sugars not only function as metabolic resources and structural constituents of cells, but also act as compatible solutes under various stress conditions [37], [40]–[42]. The salt-tolerant variety FL478 had a greater number of increased sugars, whose levels were highest at 7 d, whereas almost no sugars experienced increases at the later stress time in IR64. The heavier accumulation of metabolites at this late stage suggests their possible function as compatible solutes against osmotic challenge, a consequence of higher Na^+^ accumulation in plants after long-time exposure to salt stress.

In contrast to the consistent change patterns of AAs and sugars in both tissues, OAs decreased in leaves and increased in roots under salt stress. This opposite change pattern may be related to the different functions of leaves and roots. First, the fact that OAs decreased in leaves indicates that energy production or plant growth was repressed by salt stress. Second, the degree of cation–anion imbalance is one of the key factors determining OA levels in plants. In situations where roots take up an excess of cations, the negative charge required to restore the charge balance is often provided by OAs, such as malate, malonate, citrate and aconitate [43]–[44]. Thus, the increased OAs observed in roots may function to compensate for a charge imbalance [14], or operate as metabolically active solutes for osmotic adjustment [45]. Importantly, the larger number of more strongly and exclusively decreased OAs, including those participating in the TCA cycle, observed at early stress times (1–3 d) in FL478 compared with IR64, indicate that the growth of FL478 was depressed earlier. This result is consistent with previous observations that stomatal conductance and transpiration rate experienced greater decreases in leaves of FL478 than of sensitive variety IR29 under salt stress [18].

Several metabolites either had a common response in both genotypes or were specifically changed in FL478 during salt treatment, indicating their possible relationship to ST. In total, 6 metabolites, including two AAs (phenylalanine and threonine), one OA (citric acid) and three sugars (raffinose, melicitose and galactinol) were commonly increased in the leaves or roots of both genotypes, while 11 metabolites, including five AAs (lysine, threonine, isoleucine, proline, valine), one OA (isocitric) and five sugars (sucrose, lactose, sorbitol, mannitol and galactopyranoside), were greatly and specifically induced in leaves or roots of FL478 over the course of the treatment. Most of these metabolites are proposed to be related to stresses [40]–[41], [46]–[52]. To our knowledge, however, an association between lactose, sorbitol and melicitose and stress tolerance has not been previously reported.

Finally, the debate regarding the function of proline in stress tolerance [23], [25]–[26] can be partially resolved on the basis of our observations that leaf proline contents increased in both genotypes under stress, whereas proline content in roots decreased in IR64 but increased in FL478. We therefore conclude that proline accumulation is an indicator of stress tolerance, not an injury system. Additional longer time-series research using contrasting materials might aid assessment of conflicting opinions regarding the functions of proline in stress tolerance.

## Conclusions

We used GC-MS to comparatively analyze metabolite changes in two contrasting rice genotypes under salt stress treatment. As a result, genotype- and time-dependent metabolite profiles in response to salt stress were uncovered. First, the levels of most AAs and sugars increased consistently in both leaves and roots. In contrast, OA levels showed opposite changes between roots and leaves because of the different adaptive mechanisms of the two tissues and the differential functions of OAs against salt stress. Second, the superior ST of FL478 was attributed to its lower Na^+^ absorption and higher RWC under stress. At the metabolic level, the salt stress response of FL478 involved a greater decrease in OAs during early stress stages, with a higher accumulation of AAs and sugars, which function as effective compatible solutes against osmotic challenge, during later stress stages. Third, 11 metabolites were exclusively increased in FL478 under stress, implying their positive roles in ST. Three of these compounds—lactose, sorbitol and melicitose—are newly recognized as metabolites related to ST.

## Supporting Information

Table S1 Metabolite comparison between leaves and roots of FL478 and IR64 under control and salt stress conditions.
(XLS)

Table S2 S2.1: Results of ANOVA for genotypic differences (*G*) of metabolites in leaves and roots between FL478 and IR64 under normal growth conditions. S2.2: Results of ANOVA for tissue differences (*T*) between leaves and roots of IR64 and FL478 under normal growth conditions. S2.3: Results of ANOVA for the stability of metabolites (*t*) in leaves and roots of FL478 and IR64 under normal growth conditions.
(XLSX)

Table S3 Metabolite ratios in salt-treated FL478 and IR64 leaves compared with controls at different time points.
(XLS)

Table S4 Metabolite ratios in salt-treated FL478 and IR64 roots compared with controls at different time points.
(XLS)
